# Analysis of the Effects of *SAA1* Gene Polymorphisms on Renal Involvement in a Familial Mediterranean Fever Jordanian Population

**DOI:** 10.5152/eurasianjmed.2024.24457

**Published:** 2024-10-01

**Authors:** Ahmed Sheyyab, Rania Wahdan, Al-Ameen Al-Aitan, Mahmoud Abukhadra, Laith Hussein Ayed Naimat

**Affiliations:** 1 Department of Internal Medicine, The Hashemite University Faculty of Medicine, Zarqa, Jordan; 2 Laboratory of Genetics, Prince Hamzah Hospital, Ministry of Health, Amman, Jordan; 3 Department of Internal Medicine, Jordan University Hospital, Jordan University, Amman, Jordan

**Keywords:** Familial Mediterranean fever, serum amyloid A protein, genotypes, amyloidosis, *SAA1* polymorphisms, *MEFV* mutations

## Abstract

**Background:**

Familial Mediterranean Fever (FMF) is an inherited autosomal recessive disorder resulting from the inheritance of *MEFV* gene mutations. Patients with FMF are at increased risk of secondary amyloidosis, namely type AA. In some Mediterranean populations, the α genotype was associated with the development of renal amyloidosis, a finding not reproduced in other populations. Our study aimed to assess the association of *SAA1* genotypes with renal involvement.

**Methods:**

This is a retrospective analysis of FMF patients which were followed at our institute between January 2016 and August 2022. Familial Mediterranean Fever screening was performed using polymerase chain reaction and reverse hybridization techniques. Statistical analysis was performed using bivariate logistic regression.

**Results:**

*MEFV* analysis of the studied patients (n = 427) identified 52 patients with a homozygous genotype (12.1%) and 374 with a heterozygous genotype (87.5%). The heterozygous group were mostly heterozygous carriers of a single FMF variant (81%), while 19% were compound heterozygous. Renal involvement was revealed in 95 patients (22.2%), which were manifested as proteinuria (21.3%) and/or renal impairment in 4 patients (3%). The clinical diagnosis of amyloidosis was suspected in 6 patients only (1.4%). Analysis for *SAA1* gene genotype–phenotype correlation showed that patients with the *SAA1.1/1.1* (OR = 0.54, *P* = .452) was not statistically associated with renal involvement. Pearson Chi-square was performed to examine the association between FMF homozygosity and each *SAA1* genotype, which showed a significant association between FMF gene homozygosity with *SAA1.1/1.1* genotype (*χ* ^2^= 8.06, *P* = .018).

**Conclusion:**

In our Jordanian FMF population, we report low rates of renal involvement with a high rate of the β haplotype *(SAA1.5)*. Neither the α/α nor the β/β genotypes were associated with evidence of renal involvement.

**Main Points**

- Familial Mediterranean Fever among Jordanians carries a good prognosis with a low risk of amyloidosis (1.4%).
- In our Jordanian FMF population, the α/α genotype is not associated with higher rates of amyloidosis.
- The homozygosity rates of the *SAAα* genotype in Jordanians (14%) are similar to other Mediterranean populations but with much lower rates of amyloidosis.
- *SAAα* homozygosity seems to be more common in those with MEFV homozygotes compared to MEFV simple heterozygotes and/or compound heterozygotes.

## Introduction

Familial Mediterranean Fever (FMF) is an inherited autoinflammatory disorder that leads to episodic inflammatory reactions manifesting as periodic fevers and is distinctively associated with polyserositis. It is associated with variants of *MEFV*, which encodes pyrin, a protein expressed in granulocytes that plays a critical role in the regulation of inflammation and apoptosis.^1-3^ To date, over 393 variants of *MEFV* have been identified and documented in an online registry (https://infevers.umai-montpellier.fr/web/). Familial Mediterranean Fever is prevalent in specific ethnic groups, namely, Arabs, Jews, Armenians, and Turks.^4^ In Arabs, the FMF carrier rate is about 1 in 5.^5^

The spectrum of *MEFV* variants is distinct among the different ethnic groups. Homozygosity for the variant p.M694V has been linked to severe disease and an increased risk of renal AA amyloidosis, particularly in North African Jews,^6-8^ whereas the 2 variants p.M680I and p.M694I are found in the Arab population, generally with a low risk of amyloidosis.^9,10^ In a recent study of a North African population from Algeria, p.M694I homozygosity was shown to be associated with the development of renal amyloidosis.^11^ In this study, we will assess the distribution of *MEFV* variants in the Jordanian population and determine which variant is associated with symptomatic disease.

The phenotypic expression of FMF is likely to involve environmental factors and modifier genes, along with *MEFV* variants.^12,13^ Serum amyloid A1 is an acute-phase reactant upregulated during inflammatory FMF attacks, and its degradation products are responsible for the formation of organ fibrils and the development of secondary amyloidosis, namely type AA amyloidosis.^14-16^ *SAA1* is the most abundant isoform, and its polymorphisms might modify the disease course of FMF and the development of amyloidosis.^13,17^ Studies assessing the correlation between *SAA1* and FMF phenotypes showed contradictory results in different populations. Two Turkish studies showed that the *SAA1* α/α phenotype was associated with an increased risk of amyloidosis.^17,18^ A study conducted in Armenia demonstrated a similar trend for the same phenotype.^19^ More recently, an Algerian study showed a similar association with the α/α phenotype.^20^ However, this association was not reproduced in other populations.^21^ In Japanese patients, the γ/γ isoform was significantly associated with amyloidosis in both FMF and rheumatoid arthritis patients.^22,23^ This study aims to assess the influence of *SAA1* polymorphisms on renal involvement among our FMF patient population.

## Material and Methods

### Study Design

This retrospective study was conducted using *MEFV* testing data obtained between January 1, 2016 and August 30, 2022, at Prince Hamzah Hospital (PHH). Familial Mediterranean Fever-positive patients (n = 427) were included; these patients were either followed at PHH or referred from another facility for the purpose of *MEFV* variant testing. This study was approved by the Ethics Committee of The Hashemite University (approval number: No.8/1/2022/2023; date: October 31, 2022) which waived the need for informed consent.

### Clinical Data and Disease Complications

Clinical information and laboratory data were obtained for patients who were followed at our institute by reviewing their electronic medical records (Hakeem Electric Health Solutions™), and the following data were extracted: disease manifestations, disease severity, received treatment, and renal manifestations, including laboratory parameters of proteinuria and renal function. In cases where data were missing, the patient or the patient’s authorized representative was contacted to gather the needed information.

Familial Mediterranean Fever was diagnosed based on the FMF Liveneh diagnostic criteria.^24^ Disease severity was assessed using a disease severity score modified for pediatric FMF, where scores of 3-5 = mild, 6-–8 = moderate, and >9 = severe.^25,26^ Renal involvement was diagnosed clinically by a consultant nephrologist based on the degree of proteinuria and/or the associated decline in the glomerular filtration rate, which was calculated for adult patients only using the Chronic Kidney Disease Epidemiology Collaboration Equation.

### Screening for MEFV Variants and SAA1 Phenotypes

Identification of *MEFV *and *SAA1* genotypes was performed using PCR and reverse hybridization (FMF-SAA1 StripAssay, ViennaLab Diagnostics GmbH, Vienna, Austria). The assay tests for 12 *MEFV* variants (p.E148Q, p.P369S, p.F479L, p.M680I (G/C), p.M680I (G/A), p.I692del, p.M694V, p.M694I, p.K695R, p.V726A, p.A744S, and p.R761H) and 2 *SAA1* polymorphic loci determined the *SAA1* phenotype. *SAA1* polymorphism screening was performed for 322 patients. The identified single nucleotide polymorphisms (SNPs) *SAA1.1 *(*SAAα*),* SAA1.5 *(*SAAβ*),and *SAA1.3 *(*SAAγ*) encode Valine 52-Alanine 57, Alanine 52-Valine 57, and Alanine 52-Alanine 57 *SAA1* isoforms, respectively.^27^ Heterozygous carriers of a single variant were denoted as “simple heterozygous”, while carriers of 2 different allelic variants were denoted as “compound heterozygous”, and patients carrying 3 variants were denoted as “triple heterozygous” (1 complex allele with 2 variants and the other allele with a single variant).

### Statistical Analysis

Statistical analysis was conducted using R statistical software, version 4.3.1. Frequencies and percentages were used to report the values of categorical variables. Normally distributed data were reported as mean ± standard deviation (mean ± SD), while median and interquartile range were used to report non-normal data.

Patients were subgrouped according to the presence of *MEFV* variants and *SAA1* phenotypes. The association of genotypes with renal involvement, proteinuria, and renal impairment was assessed by conducting bivariate logistic regression. Pearson Chi-square test was used to assess the association between FMF genotype homozygosity and *SAA1* genotypes. A *P*-value of .05 was considered statistically significant.

## Results

A total of 427 patients who tested positive for *MEFV* variants were included in our study. The majority of included patients were children (69%). Of these patients, 54% were males and 47% were females. Three hundred sixty-three patients (84.4%) were symptomatic. Disease severity was categorized as mild (39.1%), moderate (36.5%), and severe (24.3%). Renal involvement was revealed in 95 patients (22.2%). This involvement was manifested as proteinuria in 91 patients (21.3%), and renal impairment in 4 patients (0.9%). Only 6 patients were clinically suspected to be diagnosed with renal amyloidosis (1.4%). Detailed demographic and clinical information for these patients is provided in Table 1.

Familial Mediterranean Fever gene mutation analysis identified 52 patients with a homozygous genotype (12.1%) and 374 with a heterozygous genotype (87.5%). The heterozygous group was mostly heterozygous carriers of a single FMF variant (78.0%). For *SAA1* gene polymorphisms, the leading genotypes were β/β (120 patients), α/β (137 patients), and α/α (45 patients). The least common genotypes were γ carrier genotypes, including α/γ (6 patients) and γ/β (14 patients). Detailed information on the genotype frequencies of *MEFV* mutations and *SAA1* gene polymorphisms is provided in Tables 2 and 3, respectively.

**Figure 1.**
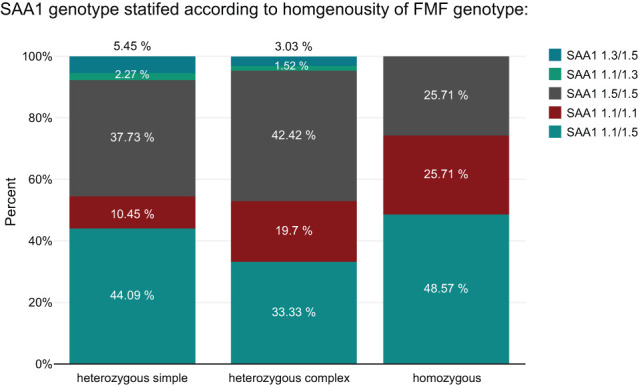
Serum Amyloid A1 genotypes pattern in the studied FMF population stratified according to homozygosity/heterozygosity status. Note that the proportion of α/α genotype carriers is the highest among MEFV carriers of the homozygous genotype (25.7%), followed by heterozygous complex (19.7%) and heterozygous simple (10.4%) genotypes.

Analysis of the associations with *SAA1* genotypes revealed that α/α genotype, compared to the non-α/α genotype group, was not statistically associated with renal involvement (OR = 0.54, *P* = .452). Table 4 demonstrates the results of statistical analyses for different genotypes compared to their non-carriers of the same genotype in respect to the presence of renal involvement. Interestingly, when comparing FMF homozygous with heterozygous groups, it showed a significant association with α/α genotype (*χ*2 = 8.06, *P* = .018), as demonstrated in Table 5. Figure 1 illustrates a stacked bar chart demonstrating the proportions of α/α genotype carriers among the included FMF population sub-grouped according to homozygous/heterozygous status.

## Discussion

Our study on the Jordanian population found that the majority were carriers of simple heterozygous mutations (67%). The remaining individuals were carriers of homozygous and compound heterozygous mutations, and some carriers of triple mutations, reflecting high carrier rates of FMF variants among our population. The carrier rate among Jordanians has been reported as one of the highest, with an approximate incidence rate of 1 in 5.^5^ In contrast, at 1.4%, renal amyloidosis was a rare diagnosis, and our finding confirms the results of previous studies conducted in Jordan, which reported an incidence of approximately 1%.^10^ This is much lower than the reported rates of amyloidosis in other populations (e.g., Turkish at 12.9%, Armenia at 24%, and Israel at 22%).^9,28,29^ Additionally, we revealed that FMF in Jordanians carries a favorable prognosis, as a low proportion of patients developed renal impairment, and none of them progressed to end-stage renal disease.

Our study of FMF-positive patients revealed important insights into the phenotypical features of the disease and its correlation with *SAA1* genotypes. Our analysis did not reveal an association between renal involvement and α/α genotypes. The literature investigating which genotypes predict the risk of amyloidosis is contradictory among different populations. Among Caucasians, multiple studies have shown an association between renal amyloidosis and the α/α genotype. A recent study of Turkish patients reported that the α/α genotype conferred a high risk of renal amyloidosis.^17^ A large study conducted in Armenia showed that the α/α genotype correlated with a higher risk of amyloidosis (OR = 6.05, *P =* .001).^19^ Also, a study from Israel showed a similar association with α/α genotype.^13^ Contradictory, a study conducted in Japan found that the γ/γ genotype was associated with a higher risk of amyloidosis.^23^ In North Africa, a recent study of an Algerian population showed that both α/α and α/γ were associated with AA renal amyloidosis.^20^ In comparison, a case–control study of Egyptian children (n = 230) found no significant differences between different *SAA1* isoforms and controls; their study was powered enough to detect differences with an effect size of 0.3.^30^ Our study, with a comparable sample size to previous studies, did not reveal an association between the α/α genotype and renal involvement.

In a broad perspective, our findings are consistent with previous studies but with some distinct differences that reflect key differences between studied populations. Firstly, renal involvement was a rare finding among our population; this finding is consistent with a previous Jordanian study which detected renal amyloidosis at 1%.^9,10^ Our measured incidence is probably an underestimate of the true incidence rate, especially considering that our study included both children and adults. However, we believe that this low rate might be explained by the high proportion of carriers with β/β and β/α genotypes among Jordanians, as the β haplotype is considered a protective allele against amyloidosis.^19,31^ Secondly, our population had slightly lower rates of α/α genotype 14%, compared to Turkish (30%), Israelis (27%), Armenians (16.9%), and Egyptians (21%).^13,18,19,30^ It is important to highlight that despite the similarities in *SAA1* genotype patterns among Jordanians compared with high-risk populations (i.e., Ashkenazi Jews), the Jordanian population has much lower rates of amyloidosis.^10^ This raises questions about whether other genetic or environmental factors play a role, especially considering that a large portion of patients with non-α / α genotypes (exceeding 70%) still develop amyloidosis. Table 6 summarizes the proportions of *SAA1* genotype comparing previous studies to our report.

Additionally, our study revealed a statistically significant association of the α/α genotype with homozygosity of the *MEFV* gene. This co-occurrence of 2 genes positioned on separate chromosomes is an unexpected finding. A plausible explanation is the occurrence of evolutionary genomic rearrangements for an unknown selective advantage in certain ethnic groups. A study comparing FMF Armenian patients in 2 neighboring countries revealed that those living in Karabakh have a major deficit of *SAA1* α homozygotes compared to those living in Armenia, which was believed to account for differences in amyloidosis rates (2.5% and 24%, respectively).^32^ Therefore, consideration the association of these genotypes is critical for the design of future genetic FMF studies.

It is important to consider other factors different between populations, especially that FMF patients with non-α/α genotype develop amyloidosis. Other genetic factors (i.e., major histocompatibility complex class-I-chain-related gene A) and environmental factors play a role.^33^ A well-designed multinational study by Touitou et al^12^ reported that country was an important risk factor for FMF severity, suggesting a possible environmental origin for amyloid susceptibility. In another study of FMF-positive Armenians, 0% of migrants living in the United States developed amyloidosis, while for FMF-positive Armenians living in Armenia, the rate exceeded 25%.^29^ We believe that future studies of FMF patients should focus on which environmental factors play a role in the pathogenesis of FMF.

Our study has limitations that need consideration. First, our analysis was based on surrogate markers (e.g., proteinuria) and did not assess renal amyloidosis through confirmatory renal or rectal biopsies. Second, the retrospective design of the study is an important limitation, inherently affected by missing data and confounding factors (e.g., colchicine intake).

Among our patient population, the frequency of the α/α genotype was similar to that in other populations, but with a much lower rate of amyloidosis. This lower rate is partly explained by the relatively higher proportion of patients who are carriers of the protective *SAA1* haplotype, specifically the β allele. Therefore, additional studies are needed to explore which factors contribute to the development of amyloidosis in a dominant portion of FMF patients who are carriers of non-α/α genotype.

## Data Availability Statement

The data that support the findings of this study are available on request from the corresponding author. Data were collected from Prince Hamzeh Hospital (PHH), from the Genetics laboratory at the Ministry of Health, Jordan.

## Figures and Tables

**Table 1.** Demographic and Clinical Features of Patients with Identified MEFV Gene Variants

Clinical Feature	Results: n = 427
**Demographic data**	
Adults/children	133/297
Male/female	223/204
Age (children)	8.2 ± 3.5
Age (adults)	33 ± 14.1
**Clinical manifestations**	
Fever	322 (75.4%)
Abdominal pain	382 (89.4%)
Pleuritis/pericarditis	109 (25.5%)
Arthritis/arthralgia	249 (58.3%)
Skin erythema	92 (21.5%)
Disease severity	
Mild (3-5)	167 (39.1%)
Moderate (6-8)	156 (36.5%)
Severe >9	104 (24.3%)
**Renal involvement** Proteinuria No proteinuria Sub-nephrotic range Nephrotic range Glomerular filtration rate (GFR)* Normal >90 mL/min Reduced <90 mL/min Renal involvement Renal amyloidosis	337 (78.9%) 85 (19.9%) 6 (1.4%) 122 (91.7%) 4 (3%) 95 (22.2%) 6 (1.4%)

*Glomerular filtration rate was assessed for adult patients only.

**Table 2.** Genotype Analysis of Patients Tested for MEFV Variants with Reporting of Genotypes Revealed with Their Frequency Rate

MEFV Genotypes					
	Homozygous n = 53		Heterozygous n = 374		
			Heterozygous Compound n = 82		Heterozygous Simple n = 292
M694V/M694V	20	V726A/M694V	14	E148Q/−	94
V726A/V726A	11	E148Q/M694V	3	V726A/−	73
M694I/M694I	10	M694V/M694I	6	M694V/−	64
E148Q/E148Q	7	M694V/R761H	2	A744S/−	16
A744S/A744S	2	M694V/M680I	4	M694I/−	11
K685R/K685R	1	V726A/M680I	13	P369S/−	11
F479L/F479L	1	M680I/M694V	1	M680I/−	9
M680I/M680I	1	M694I/V726A	10	K695R/−	9
		M694I/M680I	2	F479L/−	2
		V726A/E148Q	9	R761H/−	2
		E148Q/M694I	3	F148Q/−	1
		E148Q/M694V	2		
		E148Q/P369S	6		
		E148Q/M680I	1		
		M694I/K695R	2		
		V266A/F479L	1		
		P369S/A744S	1		
		M680I/A744S	1		
		M680/E148Q	1		

MEFV gene, Familial Mediterranean Fever gene.

**Table 3.** Genotype Analysis of Patients Tested for SAA1 Variants with Reported Frequency Rate and Percentages

SAA1 Genotypes n (%)				
α/α	α/β	β/β	γ/α	γ/β
45 (14.0)	137 (42.5)	120 (37.3)	6 (1.9)	14 (4.4)

*SAA1* gene, serum amyloid A 1 gene; α, alpha (*SAA1.1*); β, beta (*SAA1.5*); γ, lambda (*SAA1.3*).

**Table 4.** Statistical Analysis for SAA1 Polymorphisms Association between Renal Involvement Was Performed for Adult Patients Only

SAA1 Genotype	Renal Involvement		
	*P*	OR	CI
β / β	.086	2.0	0.91-4.43
α / α	.452	0.54	0.11-2.7
α / β	.066	2.2	0.95-5.1
γ / β	–	–	–
*γ* / *α*	–	–	–

The association of SAA1 genotype influence on the occurrence of renal involvement (i.e., proteinuria and/or renal impairment) was examined using logistic regression. Each genotype was compared to non-carriers of the same genotype (e.g., α/α were compared to the non-α/α group). OR, odds ratio; FMF, familial Mediterranean fever; SAA1, serum amyloid A1.

**Table 5.** Serum Amyloid A1 Genotype Was Compared Between Patients Depending on Homozygosity/Heterozygosity Status

	Heterozygous Simple n (%)	Heterozygous Complex n (%)	Homozygous n (%)	*χ* ^2^	*P*
α / α	97 (44.1)	22 (33.3)	17 (48.6)	8.06	.018
α / β	23 (10.4)	13 (19.7)	9 (25.7)	3.03	.22
β / β	83 (37.7)	28 (42.4)	9 (25.7)	2.76	.251
*γ* / *α*	5 (2.2)	1 (1.5)	–	–	–
γ / β	12 (5.4)	2 (3.0)	–	–	–

Pearson Chi-square test was performed between 3 categories: heterozygous simple, heterozygous complex, and homozygous. Our data revealed that the α/α genotype was significantly associated with the homozygosity of the FMF genotype. The co-occurrence of the 2 genes present on separate chromosomes is an unexpected finding. This finding needs further study to assess the reasons. A possible explanation is evolutionary genomic rearrangements for an unknown selective advantage.

**Table 6.** Comparison between Familial Mediterranean Fever studies of Serum Amyloid A1 Genotype Patterns in Relation to Sample Size, Homozygosity for the MEFV Gene and Ethnicity of the Studied Population

	Aysin Bakkaloglu et al	Yilmas et al	Atoyan et al	Gershoni-Baruch et al	Manal Wilson et al	Our Present Study
FMF group	n = 74	n = 50	n = 1017	n = 277	n = 105	n = 427
	Homozygous, compound heterozygous	Homozygous	Homozygous, simple heterozygous, compound heterozygous	Homozygous compound heterozygous	Homozygous compound heterozygous	Homozygous, simple heterozygous, compound heterozygous
Control group:	–	n = 50	–	–	n = 120	–
Population:	Turkish	Turkish	Armenian	Israel	Egyptian	Jordanian
SAA1 isoform:						
α/α	31%	27%	16.9%	27.30%	21.0%	14.0 %
α/β	40.5%	42%	47.3%	24.20%	35.2%	42.5 %
β/β	18.9%	22%	31.9%	35.70%	28.6%	37.3 %
*γ/γ*	1.4%	1%	–	–	–	–
γ/β	6.8%	5%	2.8%	12.6%	6.7%	4.4 %
*γ/α*	–	3%	0.8%		6.7%	1.9 %

Notice the similarity between the proportions of patients carrying the α/α genotype ranging between 21% and 31%, except for our study and the study of Armenians, which both included a larger portion of patients with a single heterozygous FMF variant.
